# Fibroblasts: invigorated targets in pre-metastatic niche formation

**DOI:** 10.7150/ijbs.87680

**Published:** 2024-01-21

**Authors:** Hongkuan Han, Cheng Qian, Mengyao Song, Chongjin Zhong, Yang Zhao, Yin Lu

**Affiliations:** 1 Jiangsu Key Laboratory for Pharmacology and Safety Evaluation of Chinese Materia Medica, School of Pharmacy, Nanjing University of Chinese Medicine, Nanjing 210023, China.; 2 Jiangsu Joint International Research Laboratory of Chinese Medicine and Regenerative Medicine, Nanjing University of Chinese Medicine, Nanjing 210023, China.; 3 Department of Biochemistry and Molecular Biology, School of Medicine & Holistic Integrative Medicine, Nanjing University of Chinese Medicine, Nanjing 210023, China.

**Keywords:** Tumor metastasis, Cancer-associated fibroblasts, Tumor microenvironment, Fibroblast-based therapy

## Abstract

At present, tumor metastasis still remains the leading contributor to high recurrence and mortality in cancer patients. There have been no clinically effective therapeutic strategies for treating patients with metastatic cancer. In recent years, a growing body of evidence has shown that the pre-metastatic niche (PMN) plays a crucial role in driving tumor metastasis. Nevertheless, a clear and detailed understanding of the formation of PMN is still lacking given the fact that PMN formation involves in a wealth of complicated communications and underlying mechanisms between primary tumors and metastatic target organs. Despite that the roles of numerous components including tumor exosomes and extracellular vesicles in influencing the evolution of PMN have been well documented, the involvement of cancer-associated fibroblasts (CAFs) in the tumor microenvironment for controlling PMN formation is frequently overlooked. It has been increasingly recognized that fibroblasts trigger the formation of PMN by virtue of modulating exosomes, metabolism and so on. In this review, we mainly summarize the underlying mechanisms of fibroblasts from diverse origins in exerting impacts on PMN evolution, and further highlight the prospective strategies for targeting fibroblasts to prevent PMN formation.

## Introduction

Widespread metastasis has constituted the primary cause of death for approximately 90% of patients with cancer^1^-^4^. It has been well accepted that metastasis involves a series of complicated processes including tumor cells penetrating into the circulation from the primary site, the formation of circulating tumor cell clusters in the circulation to escape immune eradication and flow shear forces, followed by tumor cells extravasating and colonizing in the distant organs^5^, ^6^. Given the fact that metastasis is a multi-link cascade reaction, there have been no particularly effective therapeutic strategies to fight against metastasis in the current clinical practice^7^, ^8^. Since Piget proposing the 'seed-soil' hypothesis in 1889 to Kaplan firstly defining the pre-metastatic niche (PMN) in 2005^9^, ^10^, the importance of PMN formation in propelling metastasis has been gradually appreciated and accepted, with the belief that PMN is a prerequisite for metastasis^11^, ^12^. In fact, a growing body of evidence has revealed that cytokines, exosomes and extracellular vesicles (EVs) secreted by tumor cells play pivotal roles in driving the formation of PMN and also serve as diagnostic markers for metastasis^13^-^16^. However, the evolution mechanisms of PMN are not simply associated with the cellular communication triggered by the secreted biomolecules. The contributions of other types of cells and molecular components in the tumor microenvironment (TME) and the pre-metastatic organs to PMN formation require adequate attention.

Fibroblasts are a type of cells observed in the connective tissue that are involved in a panel of critical biological processes such as wound healing and tissue repair^17^, ^18^. However, it has been increasingly appreciated that cancer-associated fibroblasts (CAFs) play diverse roles owing to the spatiotemporal heterogeneity of fibroblasts in the TME^19^. Recent studies have demonstrated that CAFs are involved in tumor proliferation, invasion, metastasis as well as drug resistance through influencing metabolism reprogramming, matrix degradation, and angiogenesis^20^-^23^. Interestingly, there have also been studies showing that the presence of α-smooth muscle actin (α-SMA) positive fibroblasts tend to exert inhibitory effect on tumorigenesis^24^. More importantly, in the process of evolution of PMN, fibroblasts serve as an emerging potential population to orchestrate the occurrence and formation of PMN. As a highly heterogeneous cell type, fibroblasts have been regarded as a promising therapeutic target to mitigate metastasis through modulating PMN formation. In this review, we emphatically summarize the latest advances on the mechanisms underlying fibroblast manipulation affects PMN formation and outline the therapeutic strategies to target fibroblasts for hindering the formation of PMN, with the purpose of providing insights into targeting fibroblasts for limiting PMN formation and metastasis.

## Role of CAFs in PMN formation

### Origin and characteristics of CAFs

In the TME, CAFs predominantly locate at the matrix of tumors and are thought to be one of the most crucial cellular components of the matrix. Intriguingly, the origins of CAFs appear to be divergent, and the current publications demonstrate that there exist four major sources for generating CAFs including (a) tissue-resident fibroblasts, (b) bone marrow-derived precursor cells and bone marrow mesenchymal stem cells, (c) cells transdifferentiated from other types of cells such as endothelial cells (ECs), vascular smooth muscle cells, pericytes, epithelial cells, adipocytes and their progenitors as well as (d) tumor cells transdifferentiated into mesenchymal cells upon epithelial-mesenchymal transition (EMT)^25^-^27^.

It has been widely held that CAFs act as a relatively abundant cell population in the TME, particular in pancreatic cancer^28^-^30^. Indeed, high heterogeneity and plasticity are deemed to be the major properties of CAFs^31^, ^32^. With the advancement of single-cell omics technology, distinct subtypes of CAFs have been identified despite that their roles in cancer progression are controversial (Table 1). Different subtypes of CAFs are characterized by distinct expression patterns of surface markers, such as fibroblast activation protein (FAP), α-SMA, fibroblast specific protein 1, platelet-derived growth factor receptor (PDGFR), etc. Of note, FAP has been recognized as a specific protein for CAFs, therefore FAP is frequently employed as a reliable marker to target CAFs in the process of cancer therapy or imaging diagnosis. Additionally, α-SMA is considered to be a solid marker for activated fibroblasts, it is commonly employed as a predominant evaluation indicator for the therapeutic outcomes of targeting CAFs^33^.

### CAFs promote tumor progression

**Figure 1:**
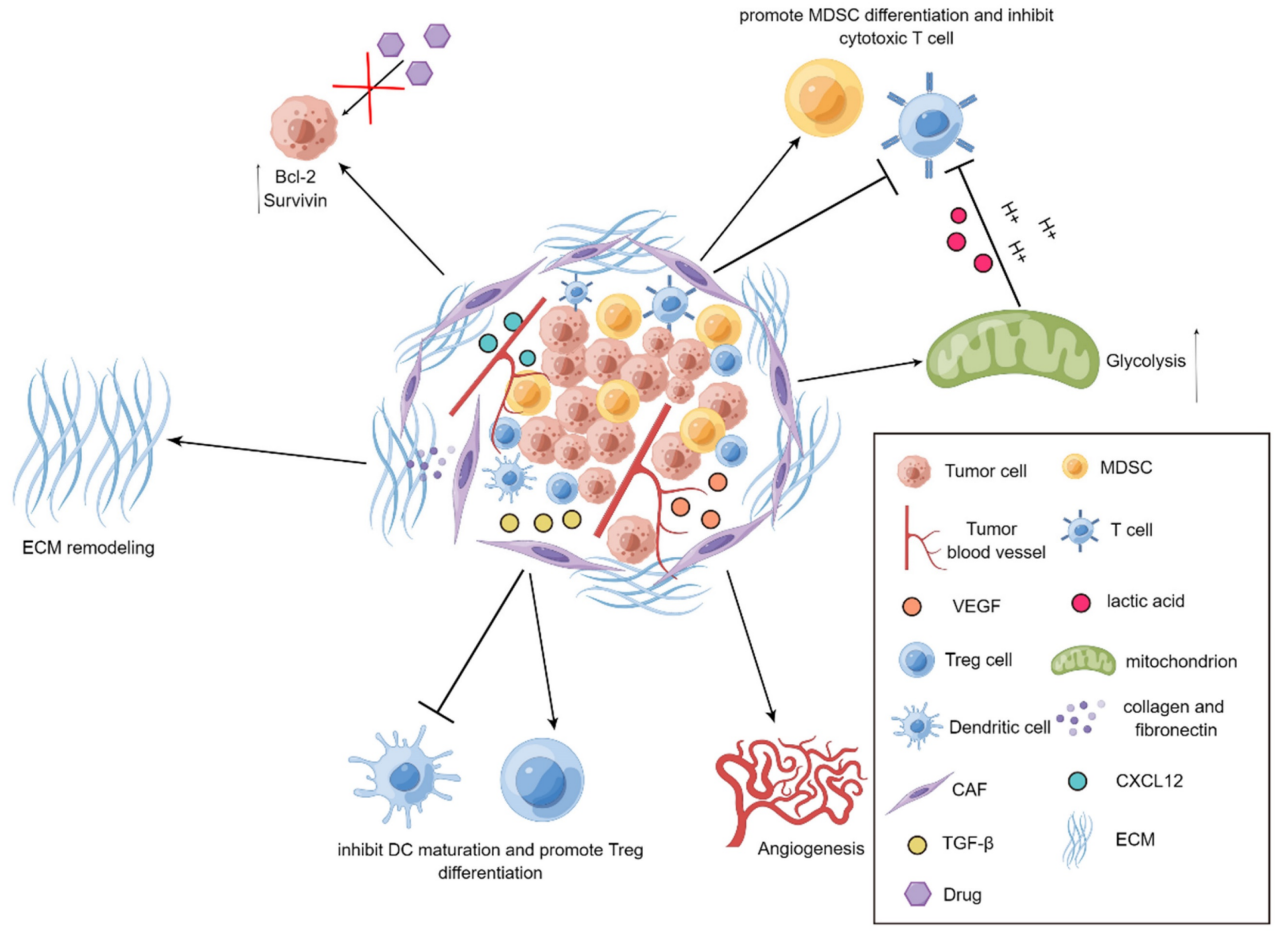
** Demonstration for roles of CAFs in tumors.** In the TME, CAFs reinforce tumor angiogenesis by releasing VEGF. CAFs produce TGF-β to repress the maturation of DCs and promote the differentiation of Treg cells. The upregulated expression of Bcl-2 and Survivin in tumor cells can be mediated through CXCL12 produced by CAFs, thereby elevating drug resistance in tumor cells. CAFs can also suppress the activity of T cells and heighten the differentiation of MDSCs. Further, the active glycolysis of CAFs increases the production of lactic acid and boosts the immunosuppression. Additionally, CAFs generate collagen and fibronectin to strengthen the remodeling of ECM.

It has been well documented that CAFs emerge as an indispensable mediator in potentiating the progression of tumors^34^-^37^. CAFs are prone to communicate with other types of stromal cells as well as tumor cells by virtue of releasing a series of fundamental cytokines, thereby repressing the functions of immune cells and exacerbating the development of tumors. For example, vascular endothelial growth factor (VEGF) derived from CAFs is essential for boosting angiogenesis to accelerate tumor progression^38^, transforming growth factor-β (TGF-β) produced by CAFs has preference to hamper the maturation of dendritic cells (DCs) and reinforce the differentiation of regulatory T (Treg) cells, and CAFs also tend to instruct the differentiation of myeloid-derived suppressor cells (MDSCs) and attenuate the activation of cytotoxic T cells through IL-6/STAT3 signaling cascade^39^, ^40^. More interestingly, CAFs are capable of secreting chemokine C-X-C motif ligand 12 (CXCL12) to elevate the expression levels of anti-apoptotic proteins Bcl-2 and Survivin in the tumor cells, leading to the occurrence of drug resistance^41^. As a robust barrier to control drug penetration and immune cell trafficking, extracellular matrix (ECM) is mainly solidified by CAFs in the TME. Through producing a large amount of collagen and fibronectin, CAFs are able to trigger the remodeling of ECM to impede the infiltration of immune cells and the penetration of anti-tumor agents into tumors, thereby assisting tumor cells to escape from the killing effects of immune cells and anti-tumor drugs^21^. Moreover, glycolysis pathway has also been observed to be highly active in CAFs in the TME. It has been uncovered that CAFs seek to discharge a large plethora of lactic acid and hydrogen ions via glycolysis route to generate an acidic TME and thus impair the activity of immune cells. Additionally, metabolites including lactate and pyruvate released by CAFs are also able to be employed as indispensable nutrients for tumor cells to facilitate tumor metabolism^42^. Further, it has been revealed that CAFs can induce the EMT of tumor cells^43^. More specifically, CAFs are prone to create a large quantity of ECM to increase the rigidity of TME and interstitial pressure, thereby exacerbating tumor invasion and metastasis^44^ (Figure 1).

In light of the unprecedented contribution of CAFs to tumor progression and metastasis, therapeutic options targeting CAFs in the TME are expected to suppress tumor development and metastasis. In fact, multiple preclinical studies have demonstrated that targeting CAFs is able to restrict tumor metastasis. For example, it has been elucidated that oroxylin A (OA), a flavonoid component from *Scutellaria baicalensis Georgi*, tends to diminish the proliferation and activation of CAFs in the MMTV-PyMT mouse breast cancer model. Mechanistically, OA can specifically bind to ACTN1 and further remarkably curtail its expression to antagonize the activation of CAFs, decline the phosphorylation of FAK and STAT3, as well as impede the secretion of chemokine C-C motif ligand (CCL2) in the CAFs to reshape the matrix microenvironment^45^. Moreover, Liu et al. found that, in a co-culture system of gastric cancer cells and CAFs derived from gastric cancer patients, the treatment of melatonin mitigated the production of metalloproteinase 2 in the CAFs to attenuate the progression of gastric cancer cells^46^. Similarly, in a co-culture system in which curcumin interfered with PC12 prostate cancer cells and CAFs derived from the prostate cancer patients, it was observed that curcumin was capable of inhibiting CAF-driven invasion of prostate cancer through modulating MAOA/mTOR/HIF-1α signaling cascade^47^. Noteworthily, the traditional drug delivery approaches still have multiple shortcomings, such as lacking drug delivery specificity and relatively easy to generate systemic toxicity, which hinder the advancement of the novel therapeutic strategies for cancer patients^48^, ^49^. Accompanying to the development of nanomedicine technology, the usage of nanocarriers enable the anti-tumor drugs to possess the properties of targeted delivery, controlled release and excellent biocompatibility. However, the current targeted therapeutic strategies for regulating CAFs still require further investigation^33^, ^50^. In particular, drug therapy and delivery options on the basis of TME are validated to be emerging in an endless stream^51^-^54^, whereas the development of drugs for limiting metastasis has been barely visualized and turns to be a research direction worthy of further exploration. In this perspective, therapeutic strategies targeting CAFs in the TME appear to be promising to govern metastasis for patients with cancer.

### Mechanisms of CAFs regulating PMN formation

A growing body of evidence has demonstrated that the evolution of PMN is able to be regulated through multifarious routes^11^, ^12^. In recent years, a large number of studies have revealed the pivotal roles of cytokines and EVs derived from tumor cells in the formation of PMN^16^, ^55^-^57^, whereas the contribution of other types of cells in the TME to PMN formation has been less illustrated. As a predominant cell component in producing ECM, an increasing number of studies have shown that CAFs also plays a key role in promoting the formation of PMN.

**Figure 2:**
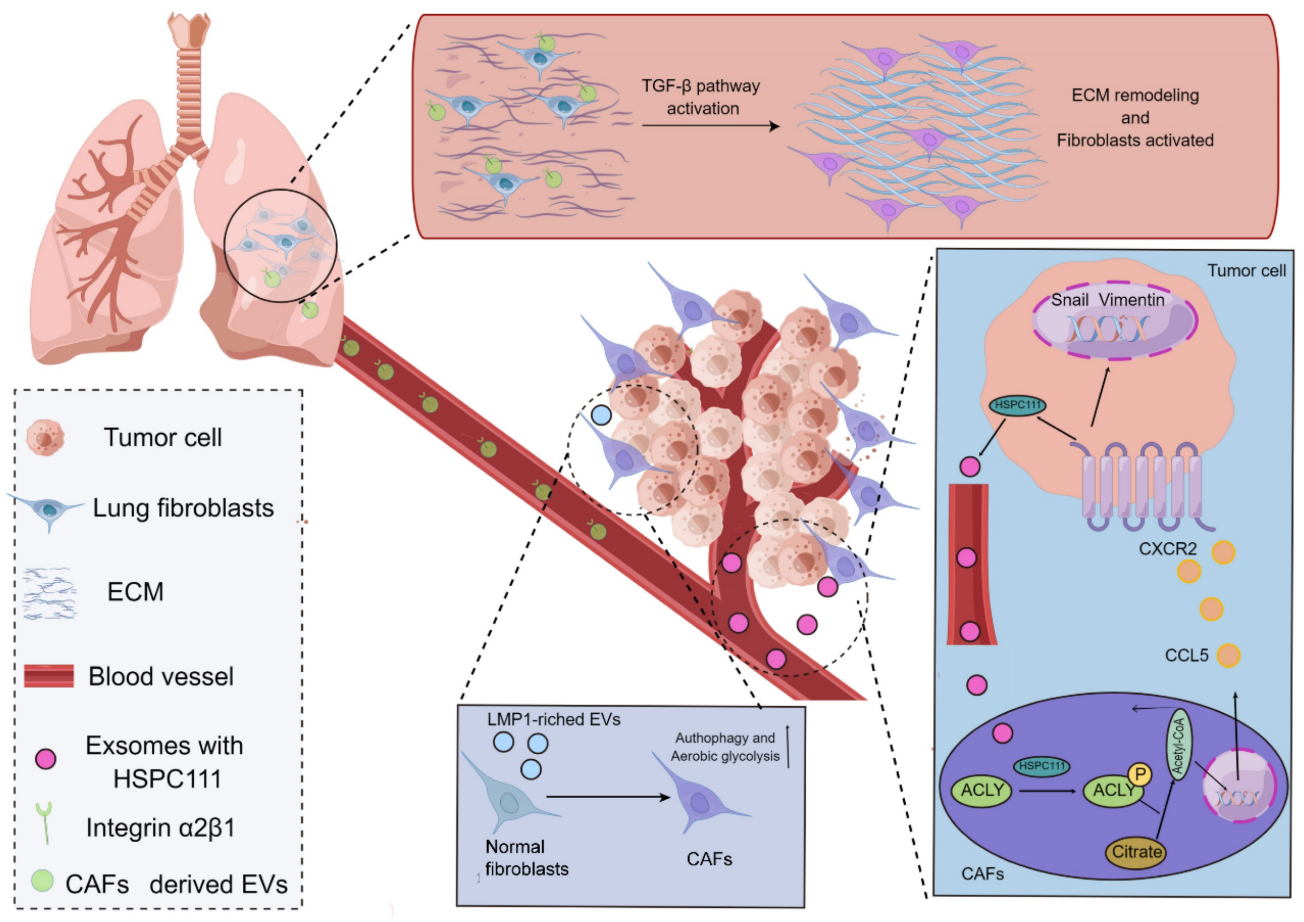
**Mechanisms of CAFs regulating the evolution of PMN.** CAFs in the TME potentiate the evolution of PMN. EVs produced by CAFs are able to express integrin α2β1 to penetrate into the metastatic organs and further lead to the activation of fibroblasts in the metastatic organs to yield the matrix remodeling and formation of PMN. The exosomes derived from cancer cells take advantage of HSPC111 to result in the activation of CAFs and the alterations of the lipid metabolism of CAFs through boosting the ACLY phosphorylation, thereby influencing the level of acetyl CoA. The accumulated acetyl CoA can improve the expression and release of CXCL5 to enhance the EMT processes. Additionally, the EVs packaging LMP1 shift normal fibroblasts into CAFs and thus enable them to present active aerobic glycolysis and autophagy.

In agreement with the functions of tumor cells, CAFs frequently play a role in the TME by secreting EVs with the purpose of building a bridge between CAFs and distant metastatic organs. As a type of substance for communications and transmission in organisms^58^-^60^, EVs exert fundamental effects on governing the biological processes of PMN formation mediated through CAFs. For instance, a study using salivary adenoid cystic carcinoma (SACC) model elucidated that the EVs derived from CAFs were capable of propelling the PMN formation in the lung tissues of SACC model, and the EVs derived from CAFs presented a stronger phenotype to give rise to matrix remodeling in the distant metastatic organs in comparison to EVs from SACC. More interestingly, a mechanistic study uncovered that integrin α2β1 was involved in mediating the uptake of EVs released from CAFs by pulmonary fibroblasts, and the inhibition of integrin α2β1 by TC I-15 retarded the formation of lung PMN and subsequent metastasis^61^. Similarly, another study highlighted that, in the nasopharynx carcinoma model, the EVs could package latent membrane protein 1 (LMP1) to transform normal fibroblasts into CAFs by virtue of the NF-κB P65 signaling cascade, and the activated CAFs were essential for promoting the proliferation and migration of nasopharyngeal carcinoma cells via aerobic glycolysis and autophagy. The PMN formation in the model was also augmented in the presence of activated CAFs^62^. Intriguingly, it has also been acknowledged that CAFs can reinforce the evolution of PMN by virtue of metabolic reprogramming, which has been substantiated by the fact that exosomes HSPC111 derived from the colorectal cancer cells tends to alter the lipid metabolism of CAFs through resulting in the phosphorylation of ATP citrate lyase (ACLY), thereby improving the expression level of acetyl CoA. The accumulate acetyl CoA further enhanced the expression and release of CXCL5 through increasing the acetylation of H3K27 in the CAFs, which led to the accelerated process of PMN formation and thus elevated liver metastasis of colorectal cancer^63^ (Figure 2).

On the basis of existing data, it has been demonstrated that CAFs are prone to accelerate the PMN formation via modulating the secretion of EVs and metabolic reprogramming. Nevertheless, since CAFs are deemed to be a population of highly heterogeneous cells^64^-^66^, focusing on their divergent phenotypes tends to be a more reasonable direction to determine the effects of CAFs on PMN formation and subsequent metastasis. Intriguingly, it has been elucidated that the interactions between tumor cells and CAFs may result in the phenotypic alterations of CAFs. More specifically, it has been revealed that miR-141, derived from ovarian cancer, has preference to target YAP1 that is the critical mediator of the Hippo signaling pathway can abate the nuclear YAP1/TAZ ratio, as well as boost the reprograming of CAFs to fibroblasts with a pro-inflammatory phenotype and propel the formation of PMN^67^.

Collectively, the interactions between CAFs and other types of cells in the TME have been revealed to exert striking effect on promoting tumor metastasis, but the specific mechanisms of action are still poorly understood. To this end, dissecting the underlying mechanisms by which CAFs interact with other types of cells in the TME to reprogram the phenotypes of PMN appears to be a promising research direction in the future. Meanwhile, although the majority of studies have illustrated the important contribution of CAFs to various characteristics of PMN formation, the EVs secreted by tumor cells need to be reshaped through ECM to influence the formation of PMN in metastatic organs. What are the underlying mechanisms by which CAFs, as important component in the ECM, transport and guide EVs into the tissues? Whether it is rational to study the functions of CAFs on transporting biomolecules as well as elucidate the mechanisms of action? In this regard, targeting EVs may be an alternative promising strategy to block the formation of PMN.

## Roles of fibroblasts of metastatic organ in PMN formation

Of note, the biological events initiated by fibroblasts in the metastatic organs for the formation of PMN are frequently ignored^68^. According to the current studies, the roles of fibroblasts in the lung stroma have been extensively explored. In this regard, we mainly concentrate on the significant contribution of lung fibroblasts to the evolution of PMN. Indeed, lung fibroblasts are thought to be the most abundant cell type in the lung stroma. The major functions of lung fibroblasts are to produce type III collagen, elastin and proteoglycan in the matrix of alveolar septa, as well as play vital roles in repairing and reconstructing lung injury. Nevertheless, aberrant proliferation and trafficking of lung fibroblasts in the stroma frequently give rise to pulmonary fibrosis, pneumonia and other types of lung-associated diseases^69^, ^70^.

In the majority types of cancers, lung is commonly the most preferential site for the malignant tumor cells to invade and metastasize^1^, ^71^. For instance, the lung metastasis rate of breast cancer has been reported to be as high as 60-70% in clinic^72^. As such, it is imperative to investigate how the cellular components in lungs modulate the formation of PMN.

### Immune remodeling of metastatic organs

On the basis of current evidence, MDSCs have always been regarded as the prominent contributor influencing the formation of PMN in the secondary metastatic organs^73^, ^74^. MDSCs stimulated by chemokines including CCL2 and CXCL2 are thought to be recruited to metastatic organs earlier than cancer cells, and they are capable of secreting cytokines, such as TGF-β, VEGF and IL-10, to give rise to immunosuppression, angiogenesis and inflammation in the process of PMN formation^75^. Noteworthily, the functions of lung fibroblasts, the "aborigines" of metastatic organs, in PMN have gradually turned to be a research hotspot, and they are usually educated by tumor-derived exosomes or EVs in the manner of “good to evil”. For example, exosomes derived from the LLC cells were co-cultured with lung fibroblasts, the release of CCL1 from fibroblast was augmented and mediated the activation of C-C motif chemokine receptor 8 (CCR8) Treg cells, thereby modulating the differentiation of Treg cells to generate an immunosuppressive microenvironment. Suppression of the CCL1/CCR8 signaling cascade with AZ084 exerted the inhibitory effect on Treg differentiation and attenuated the lung metastasis of melanoma^76^. Intriguingly, the single-cell RNA sequencing data revealed that a population of fibroblasts with specific cyclooxygenase-2 (COX-2) expression were found in the lungs of both normal and tumor-bearing mice. These fibroblasts were prone to aggravate the metastasis of breast cancer through skewing DCs and monocytes to an immunosuppressive phenotype by virtue of the release of the COX-2 mediator prostaglandin E2 (PGE-2), which could be abrogated by the specific blockade of ptgs2 (gene encoding COX-2) in the fibroblasts. Moreover, the metastasis of breast cancer was dramatically prohibited upon targeting ptgs2 transcription or pharmacological suppression of PGE2 receptor EP2/EP4 in the lung fibroblasts in combination with DC vaccines or anti-PD-1 therapy^68^.

### Inflammatory microenvironment and abnormal ECM

Indeed, the formation of inflammatory microenvironment and remodeling of ECM in metastatic organs undoubtedly provide a more fertile soil for the metastasis of tumor cells^77^-^80^. The interactions between fibroblasts and EVs in metastatic organs have been deemed to play vital roles in triggering the formation of inflammatory microenvironment and ECM remodeling. A mechanistic study demonstrated that miR-3473b as the LLC-derived exosome tended to activate NF-κB signaling in the lung fibroblasts to build up inflammatory microenvironment in lungs and further exacerbated the lung metastasis of tumors^81^. Further, it has been accepted that Cav-1 in exosomes can serve as an important signaling molecule to mediate intercellular communication. By virtue of modulating the expression levels of PMN marker genes and inflammatory chemokines in the lung epithelial cells, Cav-1 increases the secretion of tenascin-C in the lung fibroblasts, thereby promoting the deposition of ECM and suppressing the activation of the PTEN/CCL2/VEGF-A signaling pathway in the pulmonary macrophages to induce the polarization of M2 macrophages and further boost angiogenesis. In this sense, Cav-1 in exosomes is able to mediate intercellular communication and further regulate PMN before lung metastasis^82^. Likewise, the exosomes derived from melanoma contributed to the activation of p38α kinase in lung fibroblasts, which further down-regulated the downstream type I interferon signaling cascade and boosted the protein level of FAP. FAP initiated the remodeling of ECM, elevated the expression of chemokines, and recruited the infiltration of neutrophils to the lung, thereby attaining the burden of lung metastasis. Blockade of p38α expression in fibroblasts could retard the lung metastasis, indicating that p38α inhibitors might be employed as an adjuvant strategy to improve therapeutic outcomes for cancer patients with metastasis^83^. Notably, the *in vitro* experiments revealed that the EVs derived from osteosarcoma cells could increase the proliferation, differentiation as well as invasion of lung fibroblasts^84^. Additionally, Mao and colleagues demonstrated that NID1 in the EVs released by tumor cells augmented vessel permeability and triggered activation of lung fibroblasts to produce TNFR1 in the mouse liver cancer, which accelerated the pulmonary metastasis of liver cancer^85^.

It has been acknowledged that both EVs and exosomes play vital roles in the phenotypic alterations of fibroblasts, while fibroblasts have been validated to have a robust sense of "autonomy" to have impacts on the formation of PMN. Despite that it is significant to isolate the EVs or exosomes derived from tumors to determine their regulatory roles in the target cells, it is relatively difficult to completely mimic the complicated functions of PMN *in vitro*. Of note, Lin and co-workers took advantage of a co-culture system to simulate the dynamic effects of PMN. It was elucidated that breast cancer cells were capable of enabling lung fibroblasts to shift into CAFs, which in turn contributed to the recruitment of breast cancer cells^86^. Due to that the system only involved in the interactions between tumor cells and fibroblasts, it still failed to totally reflect the complexity of PMN, thus the reliable *in vitro* PMN models are still urgently required.

### Interaction with lncRNA

**Figure 3:**
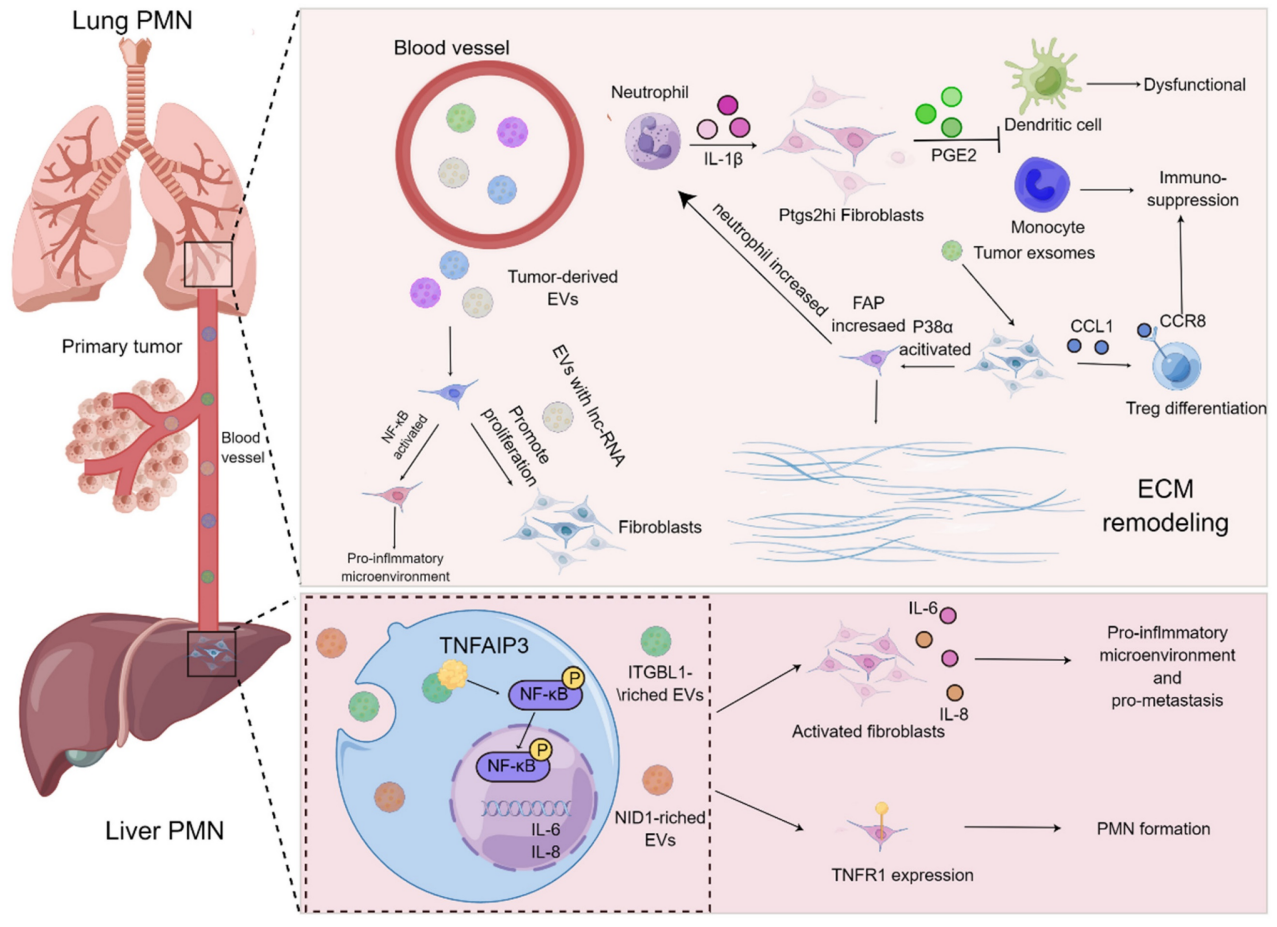
** Roles of fibroblasts of metastatic organ in PMN formation.** Tumor-derived EVs harboring LncRNAs are capable of enhancing the proliferation and activation of lung fibroblasts and generate an inflammatory microenvironment. The exosomes derived from tumors result in the activation of lung fibroblasts via the CCL1/CCR8 signaling axis to improve the proliferation of Treg cells and elevate immunosuppression. p38α signaling pathway in the lung fibroblasts is activated to augment the expression level of FAP and promote the recruitment of neutrophils and accelerate matrix remodeling for ease of PMN formation. Furthermore, IL-1β derived from neutrophils is able to boost the release of PGE2 by Ptgs2^hi^ lung fibroblast subset to trigger dysfunction of DCs and immunosuppression of monocytes. In the process of PMN in the liver, ITGBL1-rich EVs contribute to the activation of NF-κB signaling pathway of liver fibroblasts, which further augments the expression and secretion of IL-6 and IL-8 as well as propels the formation of inflammatory PMN. The EVs harboring NID1 drive PMN formation by elevating the expression level of TNFR1 in the liver fibroblasts.

Interestingly, the modulatory roles of long non-coding RNA (lncRNA) in a wealth of diseases have been well illustrated. LncRNA, with a length exceeding 200 nucleotides, is regarded as a type of RNA that cannot encode and translate into proteins and exerts a variety of functions on regulating lung metastasis of breast cancer^87^. It has been well documented that lncRNA BREA2 is able to suppress the formation of WWP2-NICD1 complex, thereby stabilizing NICD1 to strengthen the transduction of Notch signals and further accelerate the lung metastasis of breast cancer^88^. In addition, the overexpression of lncRNA ROR in the epithelial cells of breast cancer is prone to orchestrate the process of EMT through repressing the degradation of miR-205 target genes, which paves the way for exacerbating the lung metastasis of breast cancer. Of note, an increasing number of studies have also found that the aberrant expression of lncRNAs in breast cancer is closely associated with the infiltration of immune cells and subsequently influences the lung metastasis^89^. Nonetheless, there have also been a series of lncRNAs that can exert opposite effect in affecting the lung metastasis of breast cancer. For instance, lncRNA NORAD has been demonstrated to prevent the transcription of S100 family genes by virtue of prohibit the formation of YAP/TAZ-TEAD complex, thereby retarding the lung metastasis of breast cancer^90^. The interaction between lncRNA and fibroblasts has recently been clarified, high-throughput sequencing data of lncRNA in the exosomes from breast cancer have highlighted that the exosomes from breast cancer are able to promote the proliferation and migration of fibroblasts in lungs. Additionally, various lncRNA with aberrant expression are present in the lung metastatic microenvironment of breast cancer^91^ (Figure 3).

### SASP of fibroblasts in metastatic organs

Of note, cell senescence has been regarded as one of the latest properties of cancer progression^92^. It has been validated that CAFs with an aging phenotype are prone to accelerate the occurrence and development of tumors^93^-^95^. Senescence associated secretory phenotype (SASP) refers to the release of a plethora of biologically active proteins, including chemokines, cytokines, and proteases, by cells in the process of senescence to exert related biological functions^93^-^96^. It has been illustrated that the CAFs in aging skin are able to recruit immune cells via SASP to repress adaptive anti-tumor immune responses triggered by CD8^+^ T cells, thereby enhancing the growth of skin tumors^97^. In fact, recent studies have also reported the associations between fibroblasts with SASP and PMN in the lung. For instance, Huang et al^98^. uncovered that, by virtue of transcriptome and lipid metabolomics, the expression level of ACACA was markedly downregulated in lung fibroblasts, which resulted in alterations in the acetylation of protein lysine residue and fatty acid synthesis. The downregulated ACACA in lung fibroblasts contributed to senescence and inflammatory phenotypic transformation of lung fibroblasts both *in vitro* and *in vivo*. The SASP of lung fibroblasts could recruit immunosuppressive G-MDSC to the lungs by elevating the production of CXCL1.

Collectively, fibroblasts in metastatic organs passively interact with biomolecules including EVs or lncRNAs prior to exhibiting multiple pro-tumor phenotypes, which indicates that directly blocking the secretion of EVs or lncRNA may be able to reverse the abnormal phenotypes of fibroblasts at the initial stage. Notably, with the development of sequencing technology, a growing body of evidence has found a variety of spontaneously aberrant fibroblasts^99^-^101^, it is also exciting to note that targeting fibroblasts with such spontaneous abnormal phenotypes is capable of effectively limiting the occurrence and development of tumors. However, the underlying mechanisms of the formation of fibroblasts with this type of spontaneous abnormal phenotypes is still elusive. As such, multiple advanced techniques including whole-genome CRISPR screening can be employed to determine the genes associated with abnormal fibroblast formation, providing a basis for targeted therapy of abnormal-phenotype fibroblasts.

## Fibroblast based-therapies for modulating PMN formation

It has been widely held that TME is characterized by high acidity, hypoxia, and abnormal blood vessels, thereby creating the hurdle for drug delivery^102^-^105^. Furthermore, the development of therapeutic drugs for targeting PMN formation also encounters bottlenecks resulting from vessel leakage and high interstitial fluid pressure 12. The formation of PMN is featured with the increased recruitment of MDSCs, excessive angiogenesis and elevated vascular leakage. As such, the usage of antibodies to deplete myeloid cells or angiogenesis inhibitors in the mouse models has obtained great achievements on the suppression of PMN formation^106^, ^107^. Although depletion of myeloid cells is a promising strategy to prevent the formation of PMN, this approach does have various limitations. For instance, the elimination of immunostimulatory myeloid populations is capable of increasing the growth of tumors, myeloablative therapeutic options enable patients to be immunocompromised and at risk of life-threatening infections, and myeloid depletion contributes to a remarkable rebound of immunosuppressive myeloid populations derived from the bone marrow that is able to exacerbate tumor progression^108^, ^109^. Likewise, monoclonal antibody treatment also gives rise to certain side effects and toxicity^110^-^112^. Hence, the therapeutic strategies for targeting PMN are supposed to concentrate on precise targeting and low toxicity.

Recently, with the integration and application of multidisciplinary technologies, cell engineering-based therapies and targeted therapies on the basis of nanomedicines have induced markedly success for the treatment of tumor development^113^-^116^. Indeed, cell engineering-based therapies rely on immune cells and the cellular functional factors that lead to anti-tumor immunity, which can be substantiated by that CAR-T and CAR-NK therapies have been employed to execute immunotherapy in multiple types of cancers^117^, ^118^. Related studies have been conducted using cell engineering-based therapies for targeting PMN. Since it has been documented that myeloid-derived cells are prominently recruited to PMN, engineered cells with myeloid-derived cell-based gene editing secreting the immunomodulatory factor IL-12 are capable of reprograming the immunosuppressive phenotype of PMN to repress metastasis. This treatment has conferred tremendous effects on retarding rhabdomyosarcoma, but the follow-up studies still require to explore the efficacy and safety in other types of tumor models^119^. In terms of the therapeutic options of targeted drug delivery on the basis of nanomedicine, they are able to be loaded with anti-tumor agents and active molecules to facilitate the specific targeting of tumors to fight against tumor cells^120^-^122^. This strategy was validated in the retardation of PMN formation in melanoma, in which a low molecular weight heparin-tocopherol succinate nanoparticle was developed to block P-selectin/PSGL-1-triggered adhesion between myeloid-like MDSCs (G-MDSCs) and ECs to diminish the extravasation of G-MDSCs and suppress the level of matrix metalloprotein-9 (MMP-9) in the G-MDSCs^123^. Additionally, Chinese herbal medicine has also been documented to be effective on the treatment of PMN formation^124^, ^125^. For instance, Tian and colleagues revealed that the traditional Chinese medicine Baoyuan Jiedu (BYJD) Decoction could substantially impair the recruitment of MDSCs into the lungs in a mouse model of spontaneous breast cancer, which was mediated through the TGF-β/CCL9 signaling axis^126^.

Despite that the effectiveness of these therapeutic options in repressing the formation of PMN is striking, there still exist multiple limitations. Similar to the TME, PMN are commonly generated via distinct underlying mechanisms in numerous types of cancers, which frequently result in differences in the cellular and molecular components of PMNs that may influence the responsiveness of treatments^11^, ^127^. Interestingly, Wang et al. observed that a population of CD62L^dim^ expressing neutrophils were recruited in the lungs of 4T1 tumor-bearing mice, which was owing to that CXCL12 secreted from the lungs had propensity to bind to CXCR4 on the CD62L^dim^ expressing neutrophils to enable them to migrate to the lungs to generate PMN^128^. Consistently, Wu and colleagues uncovered that, in the 4T1 tumor-bearing mice without interferon alpha and beta receptor subunit 1 (Ifnar1) expression, the G-CSF level in the serum and the CXCR2 expression in the neutrophils were both increased, facilitating the recruitment of neutrophils to the lungs. In addition, the expression levels of PMN-associated proteins including Bv8, MMP-9, S100A8, and S100A9 were dramatically increased^129^. Undoubtedly, these studies emphasized the importance of neutrophils in the PMN of tumors. Furthermore, Zhang and co-workers found that, in the B16F10 melanoma model, monocyte like M-MDSCs were expanded in the circulation and lung and simultaneously released MMP-9 to promote PMN formation^130^, however, it was also shown that melanoma-derived secretions stimulated Ly6C^low^ patrol monocytes (PMo) in the bone marrow, and subsequently recruited NK cells and macrophages through PMo to kill melanoma cells in a TRAIL dependent manner. The mechanistic studies indicated that this process required the activation of nuclear receptor subfamily 4 group A member 1 (Nr4a1) transcription factor in the PMo and the secretion of PEDFs^131^. Therefore, exploring the roles of various types of cells and molecular components in PMN may pave the way for drug development.

**Figure 4:**
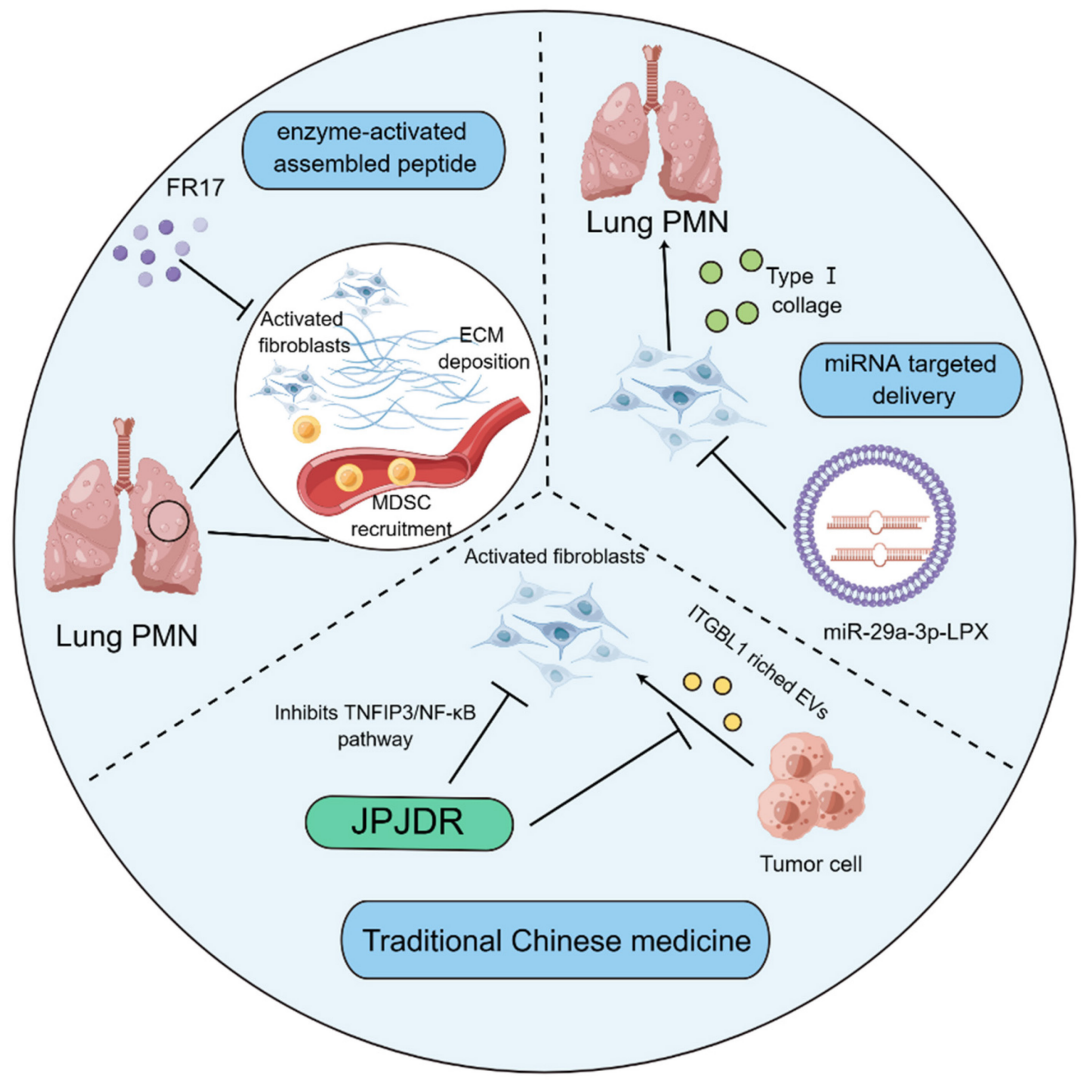
** Fibroblast-based therapies for modulating PMN formation.** The enzyme-activated assembly peptide FR17 prevents the recruitment of MDSCs through hindering the deposition of ECM and limiting the activation of lung fibroblasts, thereby retarding the production of immunosuppressive microenvironment. Liposome nano vesicles containing miR-29a-3p are able to be targeted and delivered to the lung, thereby hampering the release of type I collagen by pulmonary fibroblasts and suppressing the formation of PMN. JPJDR, a traditional Chinese medicine prescription, represses the TNFIP3/NF-κB signaling pathway to antagonize the activation of CAFs and attenuate the production of ITGBL1-rich EVs by tumor cells, thereby curbing the microenvironment to delay tumor metastasis.

Noteworthily, targeting tumor matrix has offered novel strategy for treating cancer. Given the high plasticity of CAFs, researchers seem to be more interested in therapeutic research targeting the activation of CAFs. Indeed, the internal delivery systems with nanomedicine technology are employed to prevent PMN formation through hampering the activation of fibroblasts. FR17 is an enzyme-activated assembly peptide that is capable of hindering the reshaping of the substrate tissue in the removal of the metastatic organs via antagonizing the activation of fibroblasts, thereby influencing the recruitment of MDSCs into the lungs. It has been accepted that MDSCs are able to propel the development of PMN formation through initiating the generation of immunosuppressive microenvironment. The lung-targeting drugs have the advantage of subcutaneous injection^132^. Additionally, it was reported that a lung-targeting liposomal nanovesicle containing miR-29a-3p and mimicking the exosomes was successfully established, which could diminish the collagen I of lung fibroblasts in the mouse tumor model, thereby preventing the production of PMN in lungs^133^. It has been increasingly recognized that traditional Chinese medicine therapy, as a promising therapeutic strategy^134^, has also exerted striking effects on modulating the functions of fibroblasts to limit PMN formation. JianpiJiedu Recipe (JPJDR) was documented to attenuate the activation of fibroblasts through influencing ITGBL1-rich EVs and TNFAIP3/NF-κB signaling pathway. Further animal experiments showed that JPJDR was able to lower liver metastasis of colorectal cancer (CRC) by modulating ITGBL1-rich EVs from CRC secretion, and block the ITGBL1-TNFAIP3-NF-κB signaling transmission to repress the activation of fibroblasts^135^ (Figure 4).

It has been increasingly recognized that fibroblast-based therapies appear to be more accessible to patients, more effective, and less expensive than other types of cell therapies, such as stem cell therapy^136^, ^137^. It is obvious that there are numerous approaches to intervene the process of PMN through directly targeting fibroblasts. Although engineering therapeutic strategies including nano-delivery technology and *in vivo* RNA interference technology have made unprecedented progress in precisely targeting the activation of fibroblast, the application of these engineered biological therapeutic routes is still limited due to the relatively low delivery efficiency and safety^138^-^140^. Meanwhile, the targeted delivery of nucleic acid substances including RNA *in vivo* still faces the challenge of low bioavailability^141^, ^142^, which remains an urgent issue to be addressed in future studies^143^. Moreover, traditional Chinese medicine has been utilized in the treatment of various types of diseases for thousands of years^144^, ^145^. With the advancement of modern biomedical science, the mysteries of traditional Chinese medicine with multi-target and multi-link characteristics in treating diseases have been gradually unveiled^146^. Nevertheless, owing to the multi-component nature of traditional Chinese medicine, there are still certain difficulties in investigating its specific mechanisms of action^147^. Despite that traditional Chinese medicine formulas have been elucidated to inhibit the activation of fibroblasts to prevent the progression of PMN, the specific underlying mechanisms and their components of action still remain undefined.

## Conclusion and Prospects

It has been well recognized that fibroblasts appear to be a population of cells that are sophisticated and highly heterogeneous in cancer. With the support of single cell technology, the functions of CAFs, a class of fibroblast subcluster, are being continuously evaluated. For example, Zhang et al. recently reported that CAFs expressing insulin like growth factor (IGF) 2 promoted the migration and invasion of colon cancer cells by releasing IGF2 to modulate IGF/IGFR/YAP1 signaling pathway^148^. Zhu and colleagues illustrated that CD36^+^ CAFs exhibited high levels of lipid metabolism and elevated expression of migration inhibitory factor (MIF) in the macrophages by virtue of single cell sequencing technology. CD36^+^ CAFs regulated lipid peroxidation/p38/CEBPs axis-mediated oxidative LDL uptake and were dependent on MIF expression, which contributed to the recruitment of CD33^+^ MDSCs in a MIF and CD74 dependent manner^149^. In fact, activation and reprogramming of fibroblasts have been validated to be closely associated with the formation of PMN. Nevertheless, Zeng et al. used LncRNA array to identify the differences between CAFs in breast cancer and normal fibroblasts, and it was found that LncSNHG5 was highly expressed in the CAFs. LncSNHG5 enhanced the mRNA stability of ZNF281 via binding to the m6A reader IGF2BP2. Increased ZNF281 transcriptionally regulated the expression levels of CCL2 and CCL5 to activate P38 MAPK signaling cascade in the endothelial cells to drive angiogenesis and vascular leakage during the formation of PMN^150^, all of which highlighted the possibility of CAFs independently initiating PMN formation.

Although targeting fibroblasts to prevent PMN formation is a promising strategy, unfortunately, due to the limitations of the popularization of PMN in clinical practice, there are not many targeted fibroblasts to inhibit the clinical transformation of PMN, which still requires a long exploration. However, we still believe that, in light of future studies, the advancement of multiomics technology offers a powerful tool to determine the cellular and molecular mechanisms for the occurrence and progression of tumors, which is meaningful to uncover the distinct roles of heterogeneous fibroblasts in the evolution of PMN. Furthermore, interdisciplinary technologies can also be applied to specifically target fibroblasts and actively promote the translation of the fibroblast-targeted therapies to clinical application for cancer patients.

## Funding

This work was financially supported by the projects of National Nature Science Foundation of China (82003991 and 82204687); Matching Grant of National Nature Science Foundation of China from the Nanjing University of Chinese Medicine (XPT82003991 and XPT82204687); Jiangsu Specially Appointed Professorship Foundation (013038021001). The Postgraduate Research & Practice Innovation Program of Jiangsu Province (KYCX22-2045).

## Author contributions

YL and YZ conceived and designed the study. HH and CQ wrote and finalized the manuscript. MS and CZ took part in the authoritative discussion. The final manuscript has been read and approved by all authors.

## Figures and Tables

**Table 1:** Representative makers of CAFs in different types of cancer.

CAF maker	Cancer type	Ref.
FAP	Breast, Gastrointestinal, Skin, Ovarian/Endometrial, Head and Neck, Genitourinary, Colorectal, Ductal adenocarcinoma of pancreas	151-154
PDGFRα	Breast, Gastrointestinal, Head and Neck, Genitourinary, Colorectal, Bladder	151, 155
α-SMA	Pancreatic, Colorectal, Head and neck, Lung, Melanoma, Cholangiocarcinoma, Genitourinary, Gastrointestinal, Skin	151, 155, 156
IL-6	Ductal adenocarcinoma of pancreas, Bladder, Breast	154, 155, 157
Vimentin	Breast, Gastrointestinal, Skin, Ovarian/Endometrial, Genitourinary, Pancreatic ductal adenocarcinoma	151-153
PDGFRβ	Breast, Gastrointestinal, Head and Neck, Genitourinary, Colorectal	151
CD29	Breast	151
CXCL12 (SDF-1)	Breast, Bladder, Cholangiocarcinoma, Ductal adenocarcinoma of pancreas	151, 155, 157, 158
IL-11	Colorectal, Ductal adenocarcinoma of pancreas	151, 154
FSP-1	Breast, Ovarian/Endometrial, Genitourinary, Head and Neck, Colorectal	151, 159-161
FGF-1	Ovarian/Endometrial	151
VCAM-1	Lung	151
TGF-β1	Ductal adenocarcinoma of pancreas	59
NG-2	Breast, Head and Neck	151
HGF	Cholangiocarcinoma	162

## Abbreviations

PMN: pre metastatic niche
CAFs: cancer-associated fibroblasts
EVs: extracellular vesicles
TME: tumor microenvironment
α-SMA: α-smooth muscle actin
ECs: endothelial cells
EMT: epithelial-mesenchymal transition
FAP: fibroblast activation protein
PDGFR: platelet-derived growth factor receptor
CXCL: chemokine C-X-C motif ligand
SDF: stromal cell-derived factor
CD29: cluster of differentiation 29
FGF: fibroblast growth factor
VCAM: vascular cell adhesion molecule
TGF-β: transforming growth factor-β
NG-2: Neural/Glial Antigen 2
HGF: Hepatocyte Growth Factor
VEGF: vascular endothelial growth factor
DC: dendritic cell
Treg: regulatory T
MDSC: myeloid-derived suppressor cells
STAT3: Signal Transducer and Activator of Transcription 3
Bcl-2: B-cell lymphoma-2
OA: oroxylin A
MMTV-PyMT: Mouse mammary tumor virus- Mouse mammary tumor virus
ACTN1: Actinin Alpha 1
FAK: Focal Adhesion Kinase
CCL: chemokine C-C motif ligand
MAOA: Monoamine Oxidase A
mTOR: mammalian target of rapamycin
HIF: hypoxia inducible factor
SACC: salivary adenoid cystic carcinoma
LMP: latent membrane protein
NF-κB: nuclear factor kappa-B
ACLY: ATP citrate lyase
YAP: yes-associated protein
TAZ: Tafazzin
LLC: Lewis lung carcinoma
CCR: C-C motif chemokine receptor
ITGBL: integrin beta-like
NID: Nidogen
TNFR: tumor necrosis factor receptor
COX: cyclooxygenase
PTGS: prostaglandin G/H synthase
PGE2: Prostaglandin E2
PD: programmed cell death protein
LncRNA: long non-coding RNA
SASP: senescence associated secretory phenotype
ACACA: acetyl coenzyme A carboxylase α
CAR: chimeric antigen receptor
NK: natural killer
PSGL: P-selectin glycoprotein ligand
MMP: matrix metalloprotein
S100A: S100 calcium binding protein A
TRAIL: tumor necrosis factor related apoptosis inducing ligand
PMo: patrol monocytes
PEDFs: pigment epithelial derived factors
TNFAIP: tumor necrosis factor alpha induced protein
CRC: colorectal cancer
IGF: insulin like growth factor
MIF: migration inhibitory factor
MAPK: mitogen-activated protein kinase
LDL: low-density lipoprotein
SNHG: small nucleolar RNA host gene
ZNF: zinc finger
CEBP: enhancer binding protein
Nr4a1: nuclear receptor subfamily 4 group A member 1
Ifnar1: interferon alpha and beta receptor subunit 1
NICD1: Notch1 intracellular domain
WWP2: E3 ubiquitin protein ligase 2
